# Real-Time LiDAR Point-Cloud Moving Object Segmentation for Autonomous Driving

**DOI:** 10.3390/s23010547

**Published:** 2023-01-03

**Authors:** Xing Xie, Haowen Wei, Yongjie Yang

**Affiliations:** 1 School of Information Science and Technology, Nantong University, Nantong 226019, China; 2 Department of Computer Science, Columbia University, New York, NY 10027, USA

**Keywords:** moving object segmentation, LiDAR, CNN, FPGA

## Abstract

The key to autonomous navigation in unmanned systems is the ability to recognize static and moving objects in the environment and to support the task of predicting the future state of the environment, avoiding collisions, and planning. However, because the existing 3D LiDAR point-cloud moving object segmentation (MOS) convolutional neural network (CNN) models are very complex and have large computation burden, it is difficult to perform real-time processing on embedded platforms. In this paper, we propose a lightweight MOS network structure based on LiDAR point-cloud sequence range images with only 2.3 M parameters, which is 66% less than the state-of-the-art network. When running on RTX 3090 GPU, the processing time is 35.82 ms per frame and it achieves an intersection-over-union(IoU) score of 51.3% on the SemanticKITTI dataset. In addition, the proposed CNN successfully runs the FPGA platform using an NVDLA-like hardware architecture, and the system achieves efficient and accurate moving-object segmentation of LiDAR point clouds at a speed of 32 fps, meeting the real-time requirements of autonomous vehicles.

## 1. Introduction

Presently, the key to autonomous navigation in autonomous driving systems is the ability to recognize static and moving objects in the environment, and to support predicting the future state of the environment, avoiding collisions, and planning tasks. Moving object segmentation (MOS) algorithms improve environment perception [1], localization [2], and future state prediction [3] by distinguishing between moving and static objects in 3D LiDAR point cloud data. However, the MOS task is computationally intensive and the network model is complex [4,5], so it is very important to meet the real-time processing requirements of autonomous driving applications.

Most of the existing LiDAR-based point-cloud semantic segmentation networks predict the semantic labels of point clouds, such as vehicles, buildings or roads from a single frame. However, comparing to images, LiDAR provides better object location information via consecutive frames, so that we can also use LiDAR to distinguish moving objects. Recently proposed point-based [6] or voxel-based [1] segmentation networks, although superior in performance, are structurally complicated and computationally expensive. In the recent work [4], a segmentation network based on range images was adopted, the 3D LiDAR point cloud was projected onto a 2D plane, the range images of consecutive frames were used as the intermediate representation, and a 2D convolutional neural network (CNN) was used. This network performs the moving segmentation task. Furthermore, almost all state-of-the-art point-cloud moving object segmentation networks target GPUs that may not suitable for edge computing. From a computation point of view, edge deep learning accelerators (such as NVIDIA Deep Learning Accelerator (NVDLA) [7] and Xilinx DPU [8]) do not accelerate all common operations. Therefore, designing neural networks compatible with edge deep learning accelerators is critical for real-time embedded applications.

**Figure 1:**
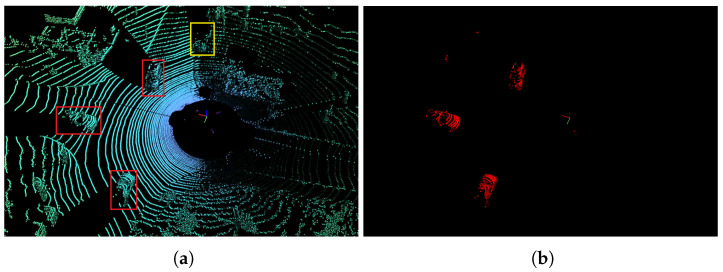
Moving object segmentation using our approach. (**a**) Raw Point Cloud; (**b**) Segmented Point Cloud.

In this paper, we propose a lightweight multi-branch network structure to solve the problem of 3D LiDAR point-cloud moving object segmentation, which can run in real time on GPU. Figure 1 shows an example scene of our segmentation, red boxes are moving cars, the yellow box is a parked car, and moving objects are represented by red masks, which also verifies the feasibility of our method. Furthermore, the MOS computing system is built for autonomous vehicles, which can perform point-cloud pre-processing and neural network segmentation. Since only post-processing steps are left to the automotive electronic Control Unit ECU, this solution significantly alleviates the computation burden of ECU, thereby reducing the decision making and vehicle reaction latency. Our moving object segmentation network achieved 32 frames per second (fps) on FPGA. The contributions of this paper are summarized below:

(1) To our knowledge, this is one of the first end-to-end FPGA implementations for a real time LiDAR point-cloud moving-object segmentation deep learning platform, a LiDAR is directly connected to the processing system (PS) side. After pre-processing, the point cloud is stored in the DDR memory, which is accessible by the hardware accelerator on the programmable logic (PL) side.
(2) A light-weight and real-time moving-object segmentation network is proposed, targeting to NVDLA. Hardware-friendly layers are used (i.e., by replacement of deconvolution with bi-linear interpolation) to greatly reduce the complexity of computation. Its IoU score on the SemanticKITTI test set is 51.3%. The inference time on NVIDIA RTX 3090TI is about 35.82 ms.
(3) An efficient moving-object segmentation network architecture is implemented on the ZCU104 MPSoC FPGA platform, which enables real-time processing at 32 frames per second (fps).

The rest of this paper is organized as follows: Section 2 summarizes the existing research results of moving-object segmentation in LiDAR point clouds and the FPGA implementation of the segmentation network. In Section 3, the proposed moving-object segmentation network model of LiDAR point cloud and its training details are described. The FPGA implementation and its results are discussed in Section 4 and Section 5, respectively. Finally, Section 6 summarizes the whole paper.

## 2. Related Work

### 2.1. LiDAR Point-Cloud Moving Object Segmentation

Existing LiDAR point cloud moving object segmentation networks can be categorized into two groups: computer-vision-based [9,10,11,12,13] and LiDAR-sensor-based [14,15,16]. However, the processing of LiDAR data remains challenging due to the uneven distribution and sparsity of LiDAR point clouds. Here, we mainly study the MOS problem of 3D LiDAR point cloud data.

In recent years, great progress has been made in semantic segmentation based on LiDAR sensor point cloud data [17,18,19,20,21,22,23,24], such as the point-cloud compression methods in [23,24] and so on. Semantic segmentation is a key step in the segmentation of moving objects in LiDAR point clouds. However, most of the existing semantic segmentation convolutional neural networks can only predict the semantic labels of point clouds, such as vehicles, buildings, and people, but cannot distinguish between actual moving objects and static objects, such as moving cars and parked ones.

The state-of-the-art scene flow method, FlowNet3D [25], is designed based on PointNet [6] and PointNet++ [26], which directly processes the original irregular 3D points without any pre-processing, and estimates each LiDAR point for two consecutive frames. The translational flow vectors include moving vehicles and pedestrians. Although these methods perform well on small point clouds, processing power becomes inefficient on larger point cloud datasets, requiring longer runtime. In addition, there are various 3D point-cloud-based semantic segmentation methods, such as SpSequencenet [27], KPConv [28], and SPVConv [29], which are also able to achieve state-of-the-art performance in semantic segmentation tasks. Among them, SpSequencenet [27] uses changes in sequence point clouds to predict moving objects. However, one problem with all networks based on operating directly on the point cloud is the dramatic increase in processing power and memory requirements, causing the point cloud to become larger. Therefore, training is difficult and cannot meet the real-time requirements of the automatic driving system.

Chen et al. [4] developed LMNet, which utilizes the residual between the current frame and the previous frame to be used as an additional input to the semantic segmentation network to achieve class-independent moving object segmentation, as well as in RangeNet++ [17] and SalsaNext [18] for performance evaluation. These networks are capable of real-time moving object segmentation running faster than the frame rate of the LiDAR sensor used. Mohapatra et al. [30] introduced a computationally efficient moving object segmentation framework based on LiDAR bird’s eye view (BEV) space. The work in [31] utilizes a dual-branch structure to fuse the spatio-temporal information of LiDAR scans to improve the performance of MOS. In contrast, Kim et al. [32] proposed a network architecture that fuses motion features and semantic features, achieving improvements in computational speed and performance metrics. In the recent work of [33], the autoregressive system identification (AR-SI) theory was used to significantly improve the segmentation effect of the traditional encoder–decoder structure, and the model was deployed in embedded devices for actual measurement.

### 2.2. FPGA Implementations of Segmentation Networks

Advanced driver-assistance systems (ADAS) are rapidly being integrated into almost all new vehicles. The LiDAR point cloud segmentation algorithm must meet the real-time requirements, which may not be well met by standard CPUs or GPUs. FPGA has the advantages of high energy efficiency ratio and flexible reconfiguration, which can realize the high energy efficiency deployment of semantic segmentation networks in ADAS. Current researchers [5,34,35,36,37,38,39] mainly study and analyze the lightweight semantic segmentation network algorithm and the accelerated computation combined with the resource characteristics of customized hardware platform. Xilinx will support Continental’s new advanced LiDAR sensor ARS 540 through the Zynq UltraScale+ MPSoC platform, partnering to create the automotive industry’s first mass-produced 4D imaging sensor, paving the way for L5-level autonomous driving systems. In Ref. [16], a LiDAR sensor is directly connected to FPGA through an Ethernet interface, realizing a deep learning platform of end-to-end 3D point cloud semantic segmentation based on FPGA, which can process point-cloud segmentation in real time.

## 3. Proposed Network

The design method of the moving object segmentation network is mainly inspired by [4]. The residual image is used as an additional input to the designed semantic segmentation network to achieve moving object segmentation. In the following, we will describe our method in detail.

### 3.1. Spherical Projection of LiDAR Point Cloud

Following previous work [16,17], we use a 2D neural network convolution to extract features from the range view (RV) of LiDAR. Specifically, we project the LiDAR point (x,y,z) onto a sphere and finally convert it to image coordinates (u,v), defined as:

(1) $$\left(\begin{array}{c} u\\ v \end{array}\right)=\left(\begin{array}{c} \frac{1}{2}\left[1-\arctan\left(y,x\right)\pi^{-1}\right]w\\ \left[1-\left(\arcsin\left(zr^{-1}\right)+f_{up}\right)f^{-1}\right]h \end{array}\right)$$

where (u,v) are the image coordinates, (h,w) are the desired range image according to the height and width, *r* represents the range of each point as $r=\sqrt{x^{2}+y^{2}+z^{2}}$, and $f=\left|f_{\mathrm{down}\;}\right|+\left|f_{up}\right|$ for the sensor’s vertical field of view.

We use Equation (1) to extract the range index *r*, 3D point coordinates (x,y,z) and intensity value *i* for each point projected to (u,v), and take them as features to be superimposed along the channel dimension. Therefore, we can directly input these features into the network, and then transform point-cloud moving segmentation into image moving segmentation.

### 3.2. Residual Images

As in ref. [4], the residual image and range view based on LiDAR point cloud are used as the input of the segmentation network, and the temporal information in the residual image is used to distinguish the static object and the pixels on the moving object, so the actual moving object and the static object can be distinguished.

Assuming that there are *N* time series of LiDAR scans in the SLAM history, $S_{j}=\left\{p_{i}\in R^{4}\right\}$ and *M* points are represented as homogeneous coordinates, i.e., $p_{i}=\left(x,y,z,1\right)$. $T_{N}^{N-1},\ldots ,T_{1}^{0}$ is denoted as the transformation matrix between N+1 scan poses, i.e., $T_{k}^{l}\in R^{4\times 4}$. Equation (2) represents the coordinate system in which the $k^{\mathrm{th}\;}$ scan transformed into the $l^{\mathrm{th}\;}$ scan

(2) $$S^{k\to l}=\left\{\prod_{j=k}^{l+1}T_{j}^{j-1}p_{i}|p_{i}\in S_{k}\right\}$$

In Ref. [4], in order to generate the residual image and fuse it into the current range image, transformation and re-projection are required. First, the transformation estimate defined in the ego-motion is compensated according to Equation (2) by transforming the previous scan to the current given local coordinate system, and next, the $S^{k\to l}$ of the past scans are re-projected to the current range image view using Equation (1). In order to calculate the residual $d_{k,i}^{l}$ for each pixel *i*, we use the normalized absolute difference between the range of the current frame and the transformed frame to calculate, as defined by

(3) $$d_{k,i}^{l}=\frac{\left|r_{i}-r_{i}^{k\to l}\right|}{r_{i}}$$

where $r_{i}$ is the range value from $p_{i}$ to the current frame at the image coordinates $\left(u_{i},v_{i}\right)$, and $r_{i}^{k\to l}$ is the range value from the transformed scan to the pixel in the same image. In the scene of moving objects, the displacement of the moving car is relatively large compared to the static background, and the residual image is obvious, while the residual image of the slowly moving object is blurred and the residual pattern is not obvious. Therefore, direct use of residual images for moving object segmentation cannot achieve good performance. Finally, we concatenate the residual image and the range view as the input of the segmentation network, and each pixel fuses the spatial and temporal information.

### 3.3. Network Architecture

**Figure 2:**
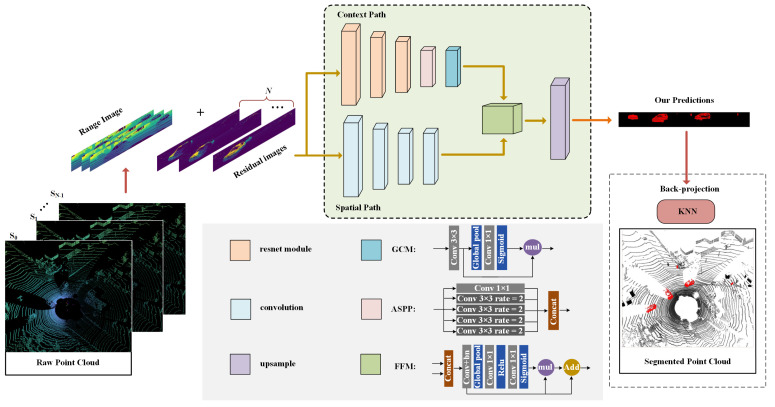
Architecture of the proposed CNN for point-cloud moving object segmentation.

In this paper, our proposed network is mainly divided into two branches: context path and spatial path, which respectively extract feature information and then fuse these feature information. The architecture of our proposed CNN for point-cloud moving object segmentation is shown in Figure 2. The backbone module utilized in the context path branch is ResNet-18 [40] for eight times fast down-sampling. Subsequently, the extracted features are fed into the Atrous Spatial Pyramid Pooling (ASPP) [41] module in order to connect features from different perceptual domains. ASPP builds convolution kernels with different receptive fields through different dilated rates to increase the receptive fields of the network and enhance the ability of the network to obtain multi-scale context, so as to obtain good performance. However, from the perspective of hardware (GPU or FPGA), dilated convolution has low efficiency and slow inference speed, and the larger the dilated rate, the longer the convolution processing time. Therefore, our network keeps the dilated rate as 2, and this method can simulate the function of ASPP without reducing the computational efficiency of GPU and NVDLA. Next, a global context module (GCM) is introduced to extract contextual information and guide feature learning of the current path. GCM consists of a global average pooling layer and a 1×1 convolution layer that extracts global context features.

The spatial path mainly retains rich spatial information to generate high-resolution feature maps, which only contains four convolutional layers. The first three convolutional layers are stride = 2, and 1/8 feature maps are extracted. The feature fusion module (FFM) [42] is used to fuse the features of context branch and spatial branch at different scales. Therefore, the features of the two channels cannot be simply weighted, but superimposed by concatenation method. FFM combines the attention mechanism for feature fusion, mainly including global pooling layer, 1×1 convolution layer, ReLU activation layer and Sigmoid layer. At the end of the network, in order to output the moving object segmentation results of the original image size, the mainstream high-performance segmentation networks, U-NET [43] and FCN [44], use layer skip connection for up-sampling. This requires a GPU or FPGA for more computation and data movement. Therefore, we up-sample the FFM output eight times using a bi-linear interpolation algorithm.

### 3.4. Training Details

We implemented a 3D LiDAR point-cloud moving-object segmentation network using PyTorch and trained on a single NVIDIA RTX 3090TI GPU. We use the method of [4] to train the network, process all point clouds according to Equations (1)–(3), and generate 64×2048 range views and residual images respectively. The residual images are then concatenated with the current range image and used as input to a 2D convolutional neural network. Trained with the new binary masks, the proposed method can separate moving and static objects label maps. During training, the network is trained with an initial learning rate of 0.01 and a weight decay of 1 × 10${}^{-4}$.

### 3.5. Dataset and Evaluation

SemanticKITTI [45] is a large-scale dataset for semantic scene understanding of 3D LiDAR point cloud sequences, including semantic segmentation and semantic scene completion. The dataset contains 28 annotated categories such as pedestrians, vehicles, parking lots, roads, buildings, etc., which further distinguishes static objects from moving objects. The raw odometry data consists of 22 sequences of point cloud data. We follow the same protocol in [17], where the sequences 00–10 are used for training and the sequence 08 is used for validation. The remaining sequences 11–21 are used as the test set. All classes are reorganized into two types: moving and non-moving/static objects according to [4]. The former one contains actually moving vehicles and pedestrians, all other classes are non-moving/static objects.

In order to evaluate the MOS performance, we follow the official guidance, using the Jaccard index or IoU [46], which is:

(4) $$\mathrm{IoU}=\frac{\mathrm{TP}}{\mathrm{TP}+\mathrm{FP}+\mathrm{FN}}$$

where TP, FP, and FN correspond to the number of true-positive, false-positive, and false-negative predictions for the moving classes.

Referring to the evaluation method proposed in [4], we use IoU to evaluate the accuracy of moving object segmentation in LiDAR point clouds. Table 1 shows the MOS performance compared to the state-of-the-art on the SemanticKITTI test set. Table 2 shows the results of the validation set. Since all operations of our network are supported by NVDLA, the network complexity is reduced and no semantic information is added. Thus, when our proposed model uses N = 8 residual images, the best performance IoU score (51.3%) is obtained, but slightly lower than the baseline LMNet [4] on the test benchmark.

**Figure 3:**
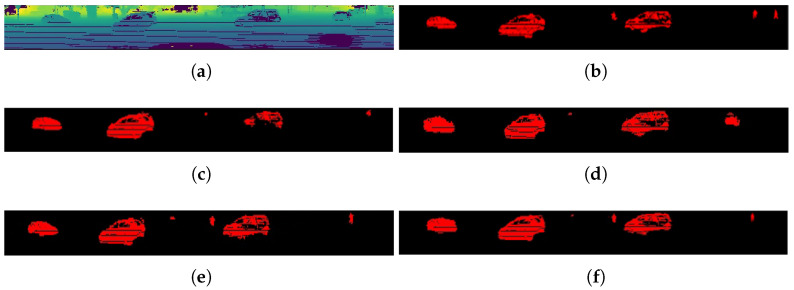
Qualitative results of different methods on the SemanticKITTI test set, where red pixels correspond to moving objects (range view images). (**a**) Range Image; (**b**) Ground Truth Labels; (**c**) BiSeNet (retrained); (**d**) SalsaNext (retrained); (**e**) LMNet; (**f**) ours.

**Figure 4:**
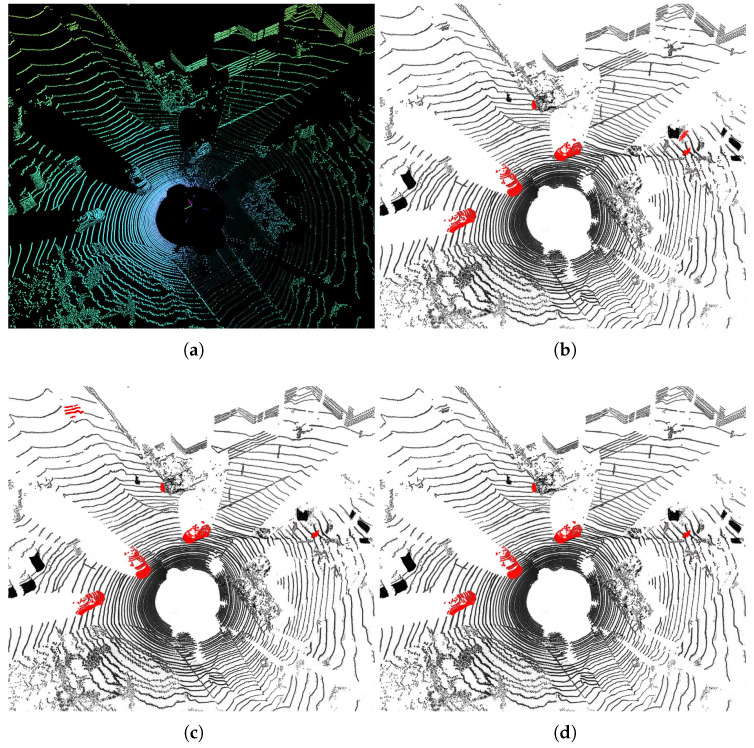
Qualitative results shown as point clouds. (**a**) Raw Point Cloud; (**b**) Ground Truth Labels, and (**c**,**d**) prediction results, where red points correspond to the class moving (**c**) LMNet; (**d**) Ours.

For qualitative evaluation, Figure 3 shows the qualitative results of different methods on the SemanticKITTI test set. Meanwhile, the qualitative results of the point clouds are shown in Figure 4. In the intersection in the figure below, there are a large number of moving objects and non-moving/stationary objects such as moving vehicles and walking people; our method can distinguish between actual moving vehicles and pedestrians, while other methods cannot detect slow-moving objects.

### 3.6. Ablation Studies

In this section, some ablation experiments on the validation set (sequence 08) of the SemanticKITTI dataset are conducted to analyze the effect of each component’s performance shown in Table 3. As shown in Table 3, it can be observed that the IoU of the dual-branch architecture can be increased by 9.6% compared to that of the single-branch architecture. On this basis, FFM, GCM, ASPP, and their combination are added. It is worth noting that the IoU of our proposed final setup can achieve 52.4%.

**Figure 5:**
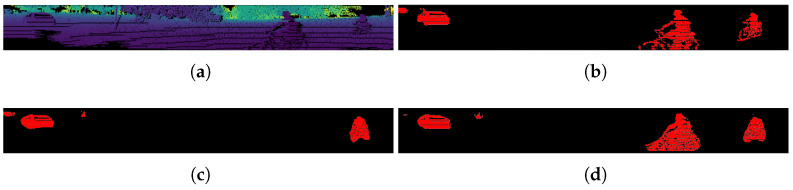
Qualitative results of different post-processing methods for MOS on the validation set, where red pixels correspond to moving objects (range view images). (**a**) Range Image; (**b**) Ground Truth Labels; (**c**) CRF; (**d**) KNN.

In our design, the K-Nearest Neighbor (KNN) post-processing is used to back-project the 2D prediction result to the 3D point cloud. In order to verify the attractive performance of the KNN post-processing in our proposed design, the comparison with regard to the back-projection between the Conditional Random Field (CRF) post-processing and the KNN post-processing is provided in Table 4. As shown in Table 4, compared to CRF post-processing, KNN post-processing results in better IoU performance. Figure 5 shows the qualitative results of different post-processing methods for MOS on the validation set. The qualitative results prove that the KNN can handle the blurred boundary of moving objects in a better way. This also obeys our expectation. Due to the fact that, during dimension reduction, different 3D points belonging to different categories might project into the same pixel in the 2D range image. Considering the principle of CRF, it contributes little to solve this issue if it is applied to a 2D range image. While KNN counts nearest points in 3D space rather than 2D.

### 3.7. Run-Time Evaluation on GPU

In autonomous driving systems, the processing speed of the moving object segmentation network must meet real-time requirements. To get a fair performance evaluation, all measurements are evaluated on the SemanticKITTI dataset 08 sequence using a single NVIDIA RTX 3090TI-24GB card and the network performances are shown in Table 5. Compared to the state-of-the-art network LMNet [4], our model clearly shows better performance, the running time is 35.82 ms, and the amount of network parameters is about 2.3 M, which is reduced to 1/3 of that in LMNet [4].

**Figure 6:**
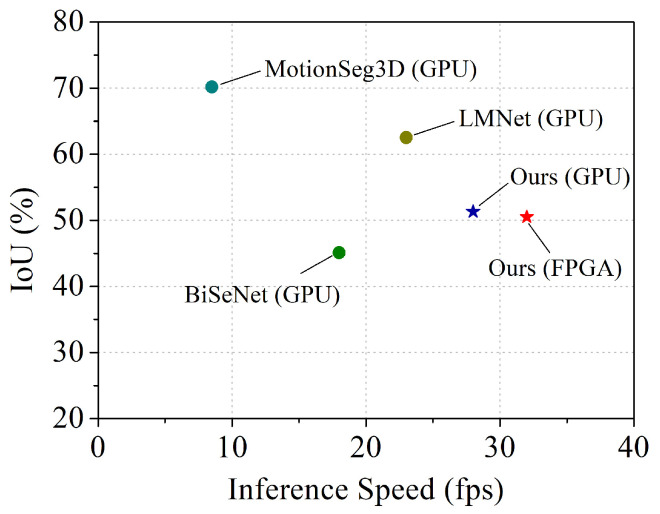
Inference speed vs. IoU on the SemanticKITTI validation set. Red star indicates our method in FPGA, the blue star indicates our method in GPU, and colored dots represent other methods.

Figure 6 shows the inference speed vs. IoU on the validation set. For practical use in embedded systems on autonomous vehicles, the IoU of our design in GPU and FPGA is sacrificed to achieving the higher inference speed compared to the other methods. Note that our implementation runs significantly faster than the 10 Hz sampling rate of mainstream LiDAR sensors [47].

## 4. Hardware Architecture

**Figure 7:**
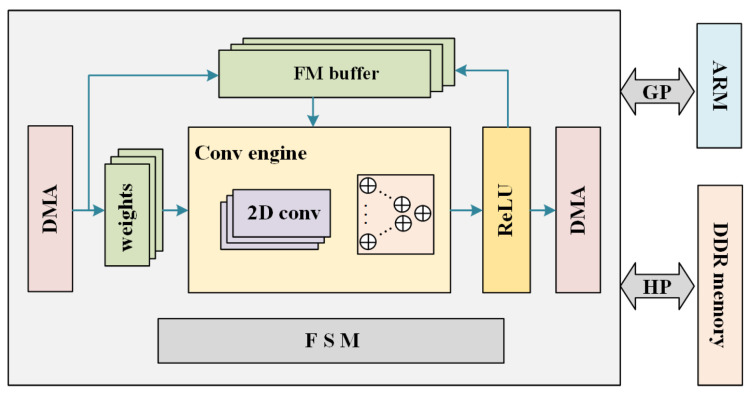
Hardware architecture of the CNN accelerator on the FPGA.

The hardware architecture of the point cloud moving object segmentation network is shown in Figure 7. It consists of processing system (ARM core) and programmable logic (FPGA) parts. ARM core is used to complete pre-processing and post-processing, such as point cloud reading, image resizing [48], result showing, etc. On the FPGA side, an NVIDIA Deep Learning Accelerator (NVDLA) like system is implemented. We tailor it and adopt it into FPGA.

The core of the convolutional engine is the MAC array. In this work, the size of the MAC array is chosen to be 32×32. To improve the processing speed and alleviate the bandwidth requirement between FPGA and DDR memory, NVDLA adopts a Ping-Pong buffer. Different from a micro-controller in NVDLA, we implement a finite state machine (FSM) to control the running order of CNN operations. In this study, we quantize the neural network to INT8 for high computation efficiency.

## 5. Results and Discussion

**Figure 8:**
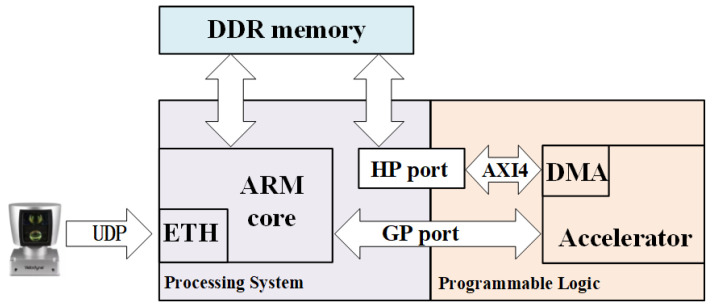
Overall system setup with LiDAR connected to the FPGA board directly.

The target hardware platform is the Zynq UltraScale+ MPSoC ZCU104 development board. Figure 8 exhibits the overall system setup with LiDAR connected to the FPGA board directly, which demonstrates our experiment setup. The LiDAR driver is implanted in the ARM processor on ZCU104 board and connected to LiDAR via UDP protocol on the Ethernet port. The ARM processor receives each set of point cloud data from LiDAR and stores it into DDR memory for NVDLA fetching.

The hardware resource usage of our proposed neural network is shown in Table 6. This design has used 91.84% of the DSP resources, if the parallelism is increased, a larger FPGA needs to be used. Table 7 shows the run-time performance of the proposed approach on SemanticKITTI Dataset. It can be observed that when running at 250 MHz, this accelerator’s processing speed is 32 fps. The estimated power consumption of the FPGA implementation is 12.8 W. The only real-time solution currently available, SalsaNext [18], runs on Nvidia Quadro P6000 GPUs and requires 600–650 W PC power support. Therefore, our solution provides a balanced and practical approach for running LiDAR point cloud moving object segmentation tasks on embedded devices. Since there are few 3D point-cloud moving object segmentation implementations on FPGA, the performance and hardware resource utilization comparison with similar works is not yet available.

## 6. Conclusions

In this study, we proposed a lightweight CNN architecture for LiDAR point-cloud moving object segmentation. Edge deep learning accelerators are designed with their limitations and computational efficiency in mind, and our proposed network structure fully supports all operations of NVDLA. On SemanticKITTI dataset, the processing time of the network on RTX 3090TI GPU is 35.82 ms and the IoU score of 51.3% is achieved. When compared to the state-of-the-art network, the network achieves a similar error performance, but using only 34% of the parameters. In addition, the proposed network successfully targets the MPSoC FPGA platform using NVDLA hardware architecture. The system successfully achieves efficient and accurate moving-object segmentation of LiDAR point clouds at 32 fps, which meets the real-time requirements of autonomous vehicles.

However, some potential problems need to be solved in the future. First of all, in order to reduce the computational complexity of our proposed network, we plan to simplify the original structure and remove the time-consuming cross-layer connections while ensuring its high performance. Secondly, due to acceleration of the model inference, the low-level details mostly sacrificed, which leads to a considerable decrease in accuracy. In view of this, the spatial path would be improved by capturing the low-level details with wide channels and shallow layers in our future works.

## Figures and Tables

**Table 1:** MOS performance compared to the state-of-the-art on the SemanticKITTI test set.

Methods	IoU (%)
SceneFlow [25]	28.7
SpSequenceNet [27]	43.2
SalsaNext [18]	46.6
LMNet [4]	62.5
BiSeNet [42]	45.1
Ours	51.3

**Table 2:** MOS performance compared to the state-of-the-art on the SemanticKITTI validation set.

Methods	IoU (%)
SalsaNext [18]	48.6
LMNet [4]	65.3
BiSeNet [42]	46.1
Ours	52.4

**Table 3:** Ablation study of components on the validation set. CP: Context Path; SP: Spatial Path; GCM: Global Context Module; FFM: Feature Fusion Module.

Methods	IoU (%)
CP	33.6
CP + SP + Sum	43.2
CP + SP + FFM	45.1
CP + SP + FFM + GCM	47.4
CP + SP + FFM + GCM+ASPP	52.4

**Table 4:** Comparison of the post-processing between the KNN and the CRF on the validation set.

	Methods	IoU (%)
(1)	KNN	52.4
(2)	CRF	49.1

**Table 5:** Run-time performance on the SemanticKITTI validation set.

	Processing Time	Speed	Parameters
BiSeNet [42]	56.3 ms	18 fps	13.76 M
LMNet [4]	42.21 ms	23 fps	6.71 M
MotionSeg3D [31]	116.71 ms	8 fps	6.73 M
Ours (GPU)	35.82 ms	28 fps	2.3 M
Ours (FPGA)	31.69 ms	32 fps	2.3 M

**Table 6:** FPGA Resource utilization for the CNN accelerator.

FPGA Resource	Used	Available	Utilization
LUT	102,707	230,400	44.58%
FF	114,221	460,800	24.79%
DSP	1587	1728	91.84%
BRAM	162	312	51.92%

**Table 7:** Run-time Performance of the Proposed Approach on SemanticKITTI Dataset.

Device	Precision	Processing time	Speed
GPU	FP32	35.82 ms	28 fps
FPGA	INT8	31.69 ms	32 fps

## Data Availability

Not applicable.
